# Calomel for Diphtheria

**Published:** 1890-04-05

**Authors:** 


### THERAPEUTICS.

### CALOMEL FOR DIPHTHERIA.

The *Cincinatti Lancet-clinic* contains some observations by Dr. J. J. Green, Pittsburg, on "Calomel in nasal diphtheria." He gave ten grains of calomel per hour, and plugged the nose with a solution of mercury bichloride, in a tampion of cotton-wool. Another physician Dr. Lange, gives infants five grains of calomal hourly, without producing salivation or purgation. It has been so universally his experience that calomel does not purge in diphtheria, that he sometimes takes this as a criterion whether the case is diphtheria or aggravated follicular tonsillitis. Another physician, Dr. McCann, has used calomel in diphtheria for years sucessfully. Notwithstanding the above we have our doubts as to this heroic use of calomel. Perhaps, however, when large doses are administered, much of it is entangled in the membrane.

